# Duodenal Neuroendocrine Tumour Presenting as GABA B Receptor Autoimmune Encephalitis

**DOI:** 10.1155/2019/3428918

**Published:** 2019-12-06

**Authors:** Dhanashree Peddawad, Manoj K. Singh

**Affiliations:** Department of Neurology, Continental Hospital, Nanakrumguda, Financial District, 500032 Hyderabad, India

## Abstract

GABA B receptor antibody positive autoimmune encephalitis is rarely seen in clinical practice. It is usually associated with small cell lung carcinoma, and occasionally with neuroendocrine tumour (NET) of lung. GABA B receptor antibody can be associated with other autoimmune antibodies like antinuclear antibody and antibody to Glutamic acid decarboxylase. We present a case with GABA B receptor autoimmune encephalitis associated with neuroendocrine tumour of the duodenum with special emphasis on correlation between immunostaining of the tumour and presence of GABA B receptor antibody.

## 1. Introduction

GABA B receptor antibody positive encephalitis other than small cell lung cancer & neuroendocrine tumour (NET) of lung has not been described in literature. Small cell lung cancer and neuroendocrine tumors have common immuno-histochemistry markers. It suggests that neuroendocrine tumors irrespective of their localization can be associated with GABA B receptor autoimmune encephalitis. It also suggests pathophysiological association of these immuno-histochemistry markers with GABA B antibody.

### 1.1. Case History

Seventy years male brought with complaints of left lower limb parasthesia of one hour duration followed by acute onset altered sensorium which started 12 hours prior to presentation to the emergency room. Altered sensorium remained for about 72 hours during hospital course from the time of onset. There were no features suggestive of motor seizures. He did not have fever, headache, vomiting, loss of consciousness, trauma, or fall.

### 1.2. Past History

**Figure 1:**
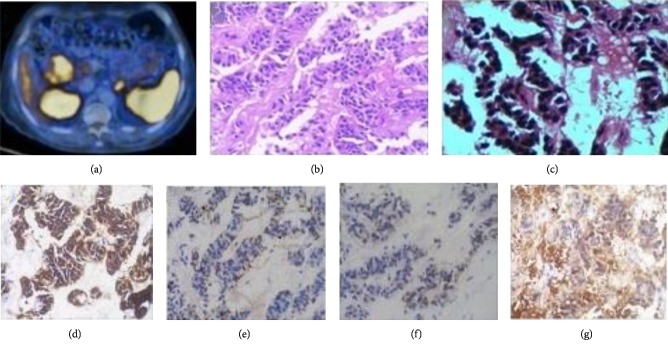
(a) Ga68DOTA-NOC—5 × 4 × 5 cm well defined DOTA avid heterogeneously intensely enhancing mass lesion in the right sub-hepatic region, abutting the proximal lateral wall of D2 segment of duodenum, situated posterior to hepatic flexure of colon s/o NET, (b) H & E Stain, (c) CT guided biopsy—Ribbons of small oval cells seperated by vascular channels. Cells have eosinophilic granular cytoplasm and relatively large dark nucleus. Cells and nuclei are uniform in size. No mitosis seen. Focal necrosis seen. Lower row images show positive stains by immunohistochemistry in our patient, (d) Synaptophysin, (e) Chromogranin, (f) CD56, and (g) Neuron specific enolase.

He was diagnosed of having hypothyroidism, hypertension, diabetes for last 15 years and Parkinson disease for last 10 years. He was receiving 50 ug of Eltroxin every day. Patient was in euthyroid state on medication. He was receiving combination of Vildagliptin and Metformin 500 mg twice a day after meals for last 6 years. Patient never had episodes of hypoglycemia in past. He was prescribed combination of Levodopa (100 mg) and Carbidopa three times in a day. His modified Hoehn and Yahr scale was 2.5. Two and half years ago, patient had 5 kg weight loss in 4 weeks associated with watery diarrhea and was diagnosed to have well differentiated para—duodenal neuroendocrine tumor. CT abdomen and pelvis then revealed 5.0 × 3.8 cms heterogeneously enhancing partially necrotic exophytic mass lesion from D2 duodenum. There was no infiltration of adjacent structures or evidence of metastasis or lymph node involvement. Whole body FDG PET scan confirmed paraduodenal mass. Ga68DOTA-NOC scan and CT guided Biopsy were also done two and half years ago. Ga68DOTA-NOC scan revealed well defined DOTA avid heterogeneously intensely enhancing mass lesion in right sub-hepatic region, abutting the proximal lateral wall of D2 segment of duodenum, situated posterior to hepatic flexure of colon suggestive of neuroendocrine tumour (Figure 1(a)). CT guided biopsy revealed well differentiated neuroendocrine tumor (Figures 1(b) & 1(c)). Immunohistochemistry stains for synaptophysin, chromogranin, CD56 and neuron specific enolase were positive. (Figures 1(d)–1(g)). Serum Chromogranin A level was 155.70 ng/mL (*N* < 39 ng/mL).

### 1.3. Clinical Findings

His vital signs including pulse rate, blood pressure, oxygen saturation were normal. Pulse rate was 84/min regular, blood pressure was 130/80 mmHg and oxygen saturation of 98% on room air. On examination he was found to have aphasia and disorientation. He could not comprehend speech of others and was repeating same words and sentences. Patient was in confusional state but was not aggressive. He could not recognize his family members. There was no cranial neuropathy. He was having mild rigidity in right upper and lower limbs. Power was normal in all limbs. Deep tendon reflexes were depressed in upper limbs and absent in lower limbs. Sensory examination could not be done as patient was not able to comprehend verbal commands. But he was responding to pain stimuli. He could stand and walk without support. Right hand mild rest tremors were present. We did not observe any intentional tremors as he tried to pick objects while carrying out examination.

## 2. Diagnostic Assessment

### 2.1. Investigations

Laboratory tests showed hemoglobin 9 gm% and ESR 70 mm/hr. Blood sugar level checked by family member at home was 240 mg/dL. Blood sugar was rechecked by emergency doctor immediately after arriving to emergency room and it was 234 mg/dL. Fasting blood glucose and glycosylated hemoglobin levels were 136 mg/dL and 7.5, respectively. Complete blood count, liver, renal function tests, electrolytes, arterial blood gas analysis, serum ammonia, thyroid function test, anti TPO antibody, Antinuclear antibody, CSF routine study and HSV PCR were normal. MRI brain did not show any significant findings. EEG showed theta background slowing and right temporal spikes.

### 2.2. Differential Diagnosis

Differential diagnosis considered in our case were acute stroke, metabolic encephalopathy including Wernickes encephalopathy & electrolyte imbalance, brain tumor, intoxication, nonconvulsive status epilepticus, viral encephalitis, thyroid autoimmune encephalitis, other autoimmune encephalitis, and paraneoplastic encephalitis. Acute stroke was ruled out as there was no diffusion restriction and angiography was normal. MRI was not suggestive of Wernickes encephalopathy. There was no space occupying lesion on MRI. There were no features of viral or limbic encephalitis on MRI brain. Thyroid antibody titers were negative. All metabolic parameters including sodium, calcium, and magnesium were within normal limits. MRI did not show any signs of anoxia or hypoglycemia. There was no history or physical findings suggestive of any toxin consumption. Possibility of nonconvulsive status epilepticus was considered. Continuous EEG monitoring done which showed occasional focal spikes in right temporal region. But absence of continuous epilptiform activity ruled out the possibility of NCSE.

In view of presence of neuroendocrine tumor, possibility of paraneoplastic autoimmune encephalitis was considered. He underwent CT chest with CT abdomen and pelvis which showed same size of paradodenal mass. CT did not reveal any lung lesion or mass.

Our case fulfilled diagnostic criteria for paraneoplastic autoimmune encephalitis proposed by Gutelkin [1]. Our case had (i) aphasia, short term memory loss and confusional state, suggesting involvement of limbic system; (ii) an interval of two & half years between onset of neurological symptoms and cancer diagnosis; (iii) exclusion of other cancer-related complications (metastasis, infection, metabolic, and nutritional deficits, cerebrovascular disorder or side-effects of therapy); (iv) EEG showing spike wave activity in right temporal lobe.

Paraneoplastic antibody panel were sent in CSF sample. Paraneoplastic antibodies Amphiphysin, CV2.1, PNMA2 (Ma2/Ta), ANNA-1/Hu, ANNA-2/Ri, PCA-1//Yo were negative. Autoimmune antibodies AMPA1 and 2, GABA B receptor antibody, LGI-1, CASPR2, and NMDA were tested using HEK293 assay in serum and CSF sample. GABA B receptor antibody was tested positive in serum. HEK293 assay is immunofluorescent method in which human embryonic kidney cells are transfected with GABA B receptor which show reactivity with CSF/serum of patient with limbic encephalitis and polyclonal antibody to GABA B receptor. Both activities are merged. Control sample does not show reactivity to patients CSF/serum sample but shows reactivity to polyclonal antibody to GABA B receptor.

GABA B receptor autoimmune encephalitis cases reported in past had similar symptoms of limbic encephalitis in elderly patients with malignancies like small cell carcinoma of lung or rarely neuroendocrine tumor of lung [2–4]. According to prior case series there are cases with normal MRI brain and acellular CSF with normal protein sugar levels [2–4].

### 2.3. Treatment

Patient did not receive any antiviral or antibiotic treatment at any point during hospital course. Patient received five doses of one gram intravenous methylprednisolone which was started 36 hours after the onset of symptoms. He stopped repeating same words or sentences and his short term memory improved. He became oriented to time place and person and started recognizing his relatives. He showed complete recovery three days after admission. Other treatment modalities like IVIG or plasma exchange were considered and discussed with patients family at the beginning of pulse therapy. As patient showed complete clinical improvement after pulse steroid therapy and did not have any recurrence, plasma exchange, or IVIG were not considered later.

### 2.4. Follow up

Patient was found symptom free during five months of follow up. Repeat GABA B receptor antibody test in serum after five months of discharge was negative. Patient was receiving tablet Imatinib 400 mg once a day and also injection Octreotide 30 mg intramuscularly once a month since last two years. Tumor symptoms were under control on this treatment. There was no watery diarrhea, weight loss, or flushing since the onset of therapy. He also did not have relapse in encephalitic symptoms after discharge.

## 3. Discussion

Our case (i) Fullfill the criteria for paraneoplastic limbic encephalitis [1]; (ii) had serum GABA B receptor antibody; (iii) with paraduodenal well differentiated neuroendocrine tumor and; (iv) showed complete recovery after IV methylprednisolone course. According to prior case series [2–5], GABA B receptor antibody encephalitis is most commonly seen in small cell carcinoma of lung. There are occasional cases of lung neuroendocrine tumor presenting as GABA B receptor positive autoimmune encephalitis. Small cell lung cancer and neuroendocrine tumors have common immunohistochemistry markers [6]. To best of our knowledge neuroendocrine tumor of gastrointestinal tract has not been seen with GABA B receptor autoimmune encephalitis. Our case of GABA B receptor autoimmune encephalitis had histologically proven duodenal neuroendocrine tumor. It suggests that neuroendocrine tumors, irrespective of their localization are associated with GABA B receptor autoimmune encephalitis. Also as per our case, histopathology of even well differentiated NET can lead to paraneoplastic manifestation. These patients may not present with obvious symptoms of malignancy and may be missed without thorough clinical and radiological evaluation. Though surgical removal is not done routinely in well differentiated neuroendocrine tumors, it is important to consider removal of tumor in case of paraneoplastic manifestations.

Small cell lung cancer and neuroendocrine tumours have common immunohistochemistry markers [6]. Our case of neuroendocrine tumour of duodenum stained positive for different immunohistochemical markers (Figures 1(d)–1(g)). Considering common immunohistochemical staining in small cell lung carcinoma and neuroendocrine tumor, it can be inferred that there is common origin of GABA B receptor antibody from one of the neuroendocrine markers like synaptophysin, chromogranin, neuron specific enolase, or CD56.
